# Cell-Penetrating Peptide Conjugates of Steric Blocking Oligonucleotides as Therapeutics for Neuromuscular Diseases from a Historical Perspective to Current Prospects of Treatment

**DOI:** 10.1089/nat.2018.0747

**Published:** 2019-02-01

**Authors:** Michael J. Gait, Andrey A. Arzumanov, Graham McClorey, Caroline Godfrey, Corinne Betts, Suzan Hammond, Matthew J.A. Wood

**Affiliations:** ^1^ Medical Research Council, Laboratory of Molecular Biology, Cambridge, United Kingdom.; ^2^ Department of Physiology, Anatomy and Genetics, University of Oxford, Oxford, United Kingdom.

**Keywords:** peptide, oligonucleotide, therapeutic, neuromuscular, conjugate

## Abstract

The review starts with a historical perspective of the achievements of the Gait group in synthesis of oligonucleotides (ONs) and their peptide conjugates toward the award of the 2017 Oligonucleotide Therapeutic Society Lifetime Achievement Award. This acts as a prelude to the rewarding collaborative studies in the Gait and Wood research groups aimed toward the enhanced delivery of charge neutral ON drugs and the development of a series of Arg-rich cell-penetrating peptides called Pip (peptide nucleic acid/phosphorodiamidate morpholino oligonucleotide [PNA/PMO] internalization peptides) as conjugates of such ONs. In this review we concentrate on these developments toward the treatment of the neuromuscular diseases Duchenne muscular dystrophy and spinal muscular atrophy toward a platform technology for the enhancement of cellular and *in vivo* delivery suitable for widespread use as neuromuscular and neurodegenerative ON drugs.

## Introduction

Therapeutic antisense oligonucleotides (ONs) of the type where the mechanism of mRNA silencing occurs through the action of the intracellular RNase H enzyme to cleave a complementary RNA strand have been reviewed extensively previously [1,2], including recently by Stanley Crooke, the recipient of the 2016 Oligonucleotide Therapeutic Society (OTS) Lifetime Achievement Award [3]. By contrast, steric blocking ONs, which are designed to bind to an RNA target and to inhibit a cellular mechanism, such as splicing, have been less well covered as therapeutics. However, we recently reviewed the chemistries used in steric blocking ON approaches [4]. Such steric blocking ONs have now emerged as approved therapeutic agents for neuromuscular diseases, such as eteplirsen for treatment of Duchenne muscular dystrophy (DMD) [5] and nusinersen (spinraza) for treatment of spinal muscular atrophy (SMA) [6]. One may expect further steric blocking ON drugs to be approved in coming years.

However there are limitations in some cases to the effectiveness of steric blocking ONs in achieving sufficient levels of activity *in vivo*, especially when they are delivered systemically and need to both penetrate the required tissues efficiently, as well as thereafter to enter cell nuclei. Thus, for example, the clinical effectiveness of eteplirsen has been questioned [5]. In addition, this drug must be used at relatively high dosage, which results in it being expensive as a therapeutic agent. The poorer intracellular efficacy of steric blocking ONs, compared to the RNase H antisense types, is partly due to their intrinsic RNA target-blocking mode of action where a competition must occur for interaction with components of the RNA recognition machinery (eg, splicing complexes). Thus this process must require at least a stoichiometric quantity of ON to reach and bind to its RNA target. By contrast, the binding of a standard antisense ON triggers a rapid cleavage of the RNA target by the cellular enzyme RNase H, which has been observed to be a much more efficient cellular process and requires lower amounts of ON to achieve an effect on the RNA target [7]. Nevertheless in certain applications, particularly in interference with nuclear events such as splicing and in targeting of toxic repetitive RNA sequences, steric blocking ONs have proved to be of great therapeutic value.

Many attempts have been made over recent years to enhance the cellular and *in vivo* delivery of ONs to improve their effectiveness, both steric blocking and otherwise. Partly this may be achieved in some cases by judicious choices of the chemistry of the backbone of ONs. For example, the use of a phosphorothioate (PS) backbone, to replace a phosphodiester backbone, results not only in a greater stability against nuclease degradation for which it was designed but also serendipitously in enhanced serum protein binding upon systemic injection, and, therefore, increases bioavailability [8,9]. Furthermore, discovery of superior entry into certain organs and cell types *in vivo*, such as liver cells, has resulted accordingly in the widespread use of the PS linkage in therapeutic antisense ONs of the RNase H-directing type.

Improvements in cell and *in vivo* delivery, as well as further increases in nuclease stability, have been obtained by incorporation of various nucleoside sugar analogs, such as 2′-*O*-methyl, 2′-*O*-methoxyethyl, and so on, reviewed in reference [4]. In addition, there have been many studies that utilize either a covalently attached ligand as delivery agent, such as the carbohydrate cluster GalNac, which has been attached to siRNA for targeting liver [10] and which also improves the potency of ON gapmers dramatically, and these are now in clinical trials [11]. By and large lipid-packaging agents have proved of less value in the case of ONs *in vivo*, as opposed to siRNA, than ligand attachment.

The emergence of a new class of peptide ligand, called a cell-penetrating peptide (CPP), has offered great opportunities for exploration of the possibilities for enhancement of delivery of an attached steric blocking ON, which was clearly needed if such ONs were to become universally accepted as efficient drugs [12]. This review concentrates initially on the contributions of the Gait group in ON and conjugate synthesis toward the award of the 2017 OTS Lifetime Achievement Award as a prelude to the exciting collaborative work of the Gait and Wood groups in the development of peptide-ON conjugates as therapeutics in treatment for neuromuscular diseases.

## RNAse H and Initial Steric Blocking Activities of Antisense ONs

The Gait group worked on the development of methods of oligodeoxynucleotide synthesis on solid support from 1975 [13] to 1984 [14], which was superseded by the outstanding phosphoramidite chemistry of Caruthers and colleagues [15] (OTS Lifetime Achievement Award winner of 2018) and automated by Applied Biosystems and other companies. Initial pioneering antisense experiments of Zamecnik and Stephenson were designed to be by a steric blocking mechanism [16], but later such ONs were found to utilize an RNase H directing mechanism of action to cleave a bound complementary RNA strand, reviewed in reference [3].

The field of therapeutics took a major step forward in the mid 1980s through the chemical synthesis of PS internucleotide linkages in ONs, based on the pioneering work of Fritz Eckstein (recipient of the 2015 OTS Lifetime Achievement Award [17], where a simple sulfur atom replaces an oxygen atom). PS-linked ONs are much more resistant to nuclease degradation than phosphodiesters, and thus, cellular activities were found to be much higher. PS ONs were later found to be active in *in vivo* models and quickly became the primary choice for therapeutics development. Initially the concept was to use ONs to block translation of mRNA, but in due course it became clear that the concentrations of such ONs needed for activity were significantly larger than for RNase-H-utilizing ONs [7]. In the years that followed most of the development of therapeutic ONs took place using gapmers, where a central core of deoxynucleotides containing PS linkages was flanked by a number of 2′-modified nucleotide analogs on each arm, such analogs being insensitive to recognition by RNase H. This also results in an increase in the binding strength of such ONs, since RNA:RNA duplexes are stronger than DNA:RNA duplexes, and also the protection of the ON ends from nuclease degradation. This configuration still allowed the cleavage by RNase H of the RNA complementary to the central core of the ON. Subsequently chemists developed many nucleotide analogs suitable for the gapmer flanks [7].

However the original concept of steric blocking started to be explored more through the use of ONs that were fully modified by the inclusion of such analogs at every position, such that the ONs are no longer able to direct RNase H cleavage. While the new therapeutics companies concentrated on development predominantly of gapmers, scientists looked to understand which are the optimal intracellular activities for steric blocking ONs [18,19].

**FIG. 1.**
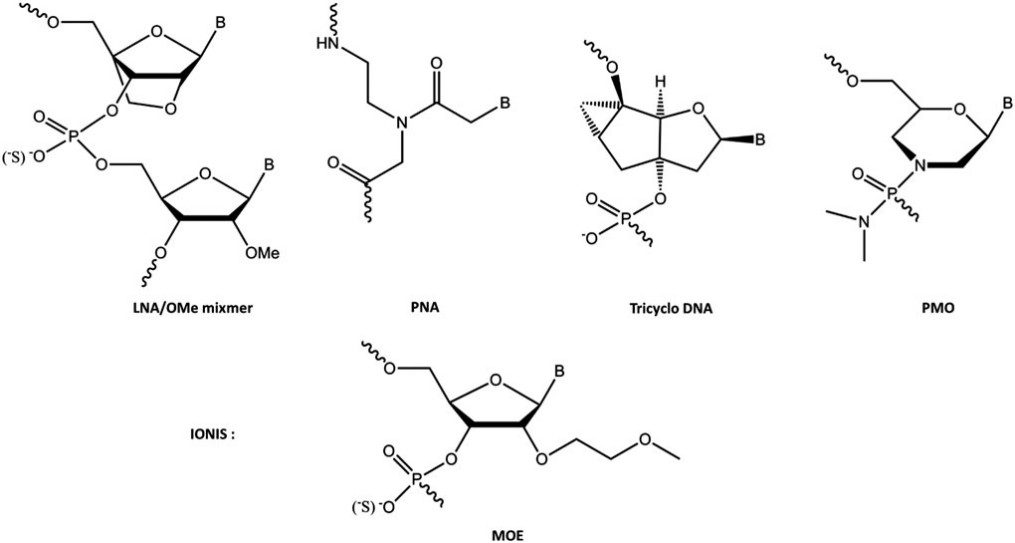
Nucleotide analogs used by the Gait group for studies of steric block ON activities in comparison to 2′-MOE nucleotides used in the Ionis research studies. ON, oligonucleotide.

At MRC-LMB in the Gait laboratory, in collaboration with the Karn laboratory, the protein-RNA interactions involved in *trans*-activation by the HIV-1 *trans*-activator protein Tat were elucidated as a potential anti-HIV drug target [20]. Then in collaboration with Wengel and colleagues in Denmark, ON inhibition studies were explored by use of certain 2′-modified ONs as mixmers, in particular mixmers of 2′-*O-*methyl (OMe) and Locked Nucleic Acids (LNAs) (Fig. 1), for the steric blocking inhibition of the *trans*-activation responsive RNA (TAR) stem-loop region of HIV-1 that binds the HIV-1 Tat protein. By use of a HeLa cell line containing stably integrated plasmids expressing firefly luciferase under HIV-LTR/Tat dependence, as well as a *Renilla* luciferase constitutive control, submicromolar, selective, dose-dependent, and sequence-dependent intracellular inhibition of Tat-TAR *trans-*activation by a 12-mer mixmer ON was shown [21]. No intracellular activity was observed for the corresponding OMe 12-mer. The ON length could be reduced to 12 because of the tight binding to complementary RNA of the included LNA analogs, but a 12-mer containing 11 contiguous LNA residues was less effective than a mixmer both in binding TAR and in inhibition of Tat-dependent transcription, probably because the latter is more flexible [21]. This was the first time that intranuclear cell activity had been demonstrated for a steric blocking ON.

In collaboration with Leumann and colleagues and Toulmé and colleagues, a 16-mer tricyclo ON (Fig. 1), although binding to TAR RNA more weakly, was found to be as good as a 16-mer OMe/LNA mixmer ON in suppressing Tat-dependent *in vitro* transcription in HeLa cells when delivered by cationic lipid. No inhibition was observed in the HeLa cell reporter model for tricyclo-DNA/OMe mixmers, even though their affinities to TAR RNA were strong and their cell distributions did not differ from ONs containing all or predominantly tricyclo-DNA residues. A tricyclo-DNA 16-mer showed sequence-specific inhibition of β-galactosidase expression in an anti-HIV HeLa cell reporter assay [22]. Thus intranuclear cell activity was not confined to LNA/OMe mixmers, but might be achievable with a range of ON analog types.

## Cell Delivery by Conjugates of ONs with CPPs

It was clear that, to obtain nuclear activity with LNA/OMe ONs required for inhibition of Tat-dependent *trans*-activation, it was necessary to formulate the steric blocking ON with a cationic lipid delivery agent. As a more practical alternative to avoid the use of complex formulation strategies and which might be more appropriate for therapeutic development, certain covalently attached CPPs [23] were investigated to deliver steric blocking LNA/OMe ONs into the nucleus of cells to inhibit Tat-dependent *trans*-activation [24]. Although uptake into the cell nucleus had been observed with fluorescent ON-CPP conjugates previously, reviewed in reference [25], many of such results were likely to have been compromised by the use of strong cell fixatives that altered the cell distribution of CPPs during such use [26].

Thus to avoid the potential for fixation artifacts, we utilized nonfixed cells to demonstrate enhanced cellular uptake. However, disulfide-linked conjugates of a 5′-thiol substituted 12-mer LNA/OMe mixmer ON targeting the TAR RNA stem-loop with the well-known cationic CPPs, HIV-1 Tat (48–58) peptide, Penetratin, or R_9_F_2_ was only seen in cytosolic vesicles when incubated with Tat-TAR reporter HeLa cells, suggesting nuclear exclusion. No inhibition of Tat-dependent *trans*-activation activity was seen unless cationic lipofection was used for delivery [27]. Uptake only into human fibroblast cytosolic compartments was seen for Tat, Penetratin, R_9_F_2_, and Transportan conjugates. Large enhancements of HeLa cell uptake into cytosolic compartments were seen when free Tat peptide was added to the Tat conjugate of 12-mer OMe/LNA ON or the Penetratin peptide to the Penetratin conjugate of the same ON, suggesting the possibility that delivery activities observed by others may have been due to a packaging effect of the use of excess of these peptides [27].

## CPP Conjugates of Charge Neutral PNA ONs

**FIG. 2.**
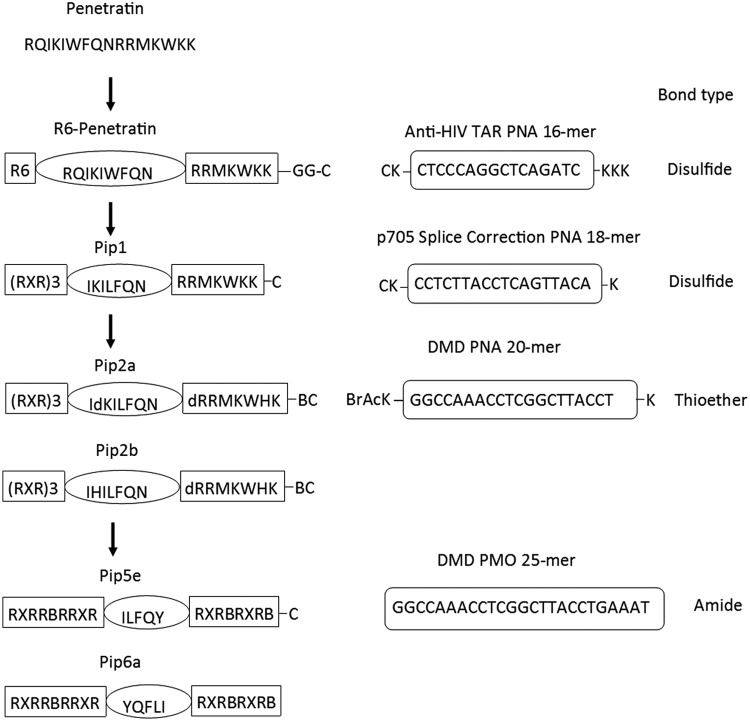
Development of novel Pip sequences starting from the CPP Penetratin. These include on the *left* side R_6_-Penetratin (R6-Pen), Pip1, which was the initial peptide disulfide linked to PNA 705 for splicing redirection, Pip2a and Pip2b, the initially protease stabilized peptides, as well as the more recent Pip5e and Pip6a. PMO sequences and their linkage functionalities are shown on the *right* side. R6-Pen and early Pip were conjugated to an anti-TAR PNA or a splice-correcting PNA705 through a disulfide linkage, whereas later Pip were either stably thioether linked to PNA705 or amide linked to PMO for exon skipping in DMD *mdx* cells. CPP, cell-penetrating peptide; DMD, duchenne muscular dystrophy.

**FIG. 3.**
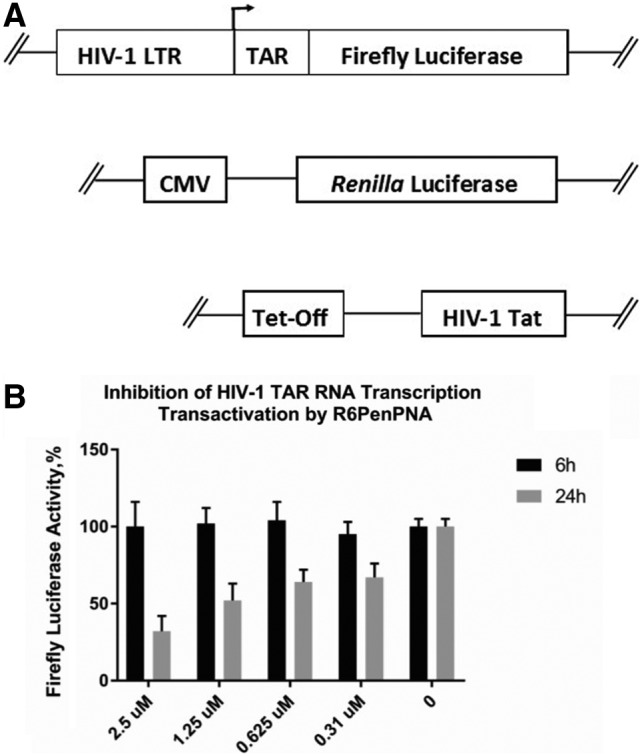
**(A)** The HIV-1 *trans*-activation assay; **(B)** inhibition of HIV-1 TAR RNA *trans*-activation by R6Pen-PNA as measured by the reduction of firefly luciferase activity.

To find ON analogs that may enter cells and be delivered to the nucleus more easily, attention was therefore turned to the use of CPP conjugates of charge-neutral ONs and, in particular, PNA ONs (Fig. 1). In this study, by contrast, much greater success in nuclear delivery was observed when certain CPPs were disulfide linked to a 16-mer charge neutral PNA targeting the TAR RNA stem loop. Disulfide-linked PNA conjugates of two types of CPP [Transportan or a novel chimeric peptide R_6_-Penetratin, where six Arg residues were added to the N-terminus of the well-known Penetratin peptide (Fig. 2)] exhibited dose-dependent inhibition of Tat-dependent *trans*-activation in the Tat-TAR dependent reporter HeLa cell assay when incubated for 24 h (Fig. 3). Activity was reached within 6 h if chloroquine, an endosomal release agent, was coadministered (data not shown). While some nuclear uptake was observed in nonfixed HeLa cells following incubation of fluorescein-labelled Transportan or R_6_-Penetratin conjugated PNA, the majority of fluorescent signal was observed in cytosolic vesicles. Interestingly, stably linked conjugates of Tat and Transportan (or TP10, a shorter Transportan analog) with PNA were inactive when delivered alone, but attained *trans*-activation inhibition only in the presence of chloroquine [28]. Thus although it was possible to obtain nuclear activity for CPP-PNA conjugates, the major part of such conjugates was seen to be located in cytosolic vesicles.

## A Splicing Redirection Assay

**FIG. 4.**
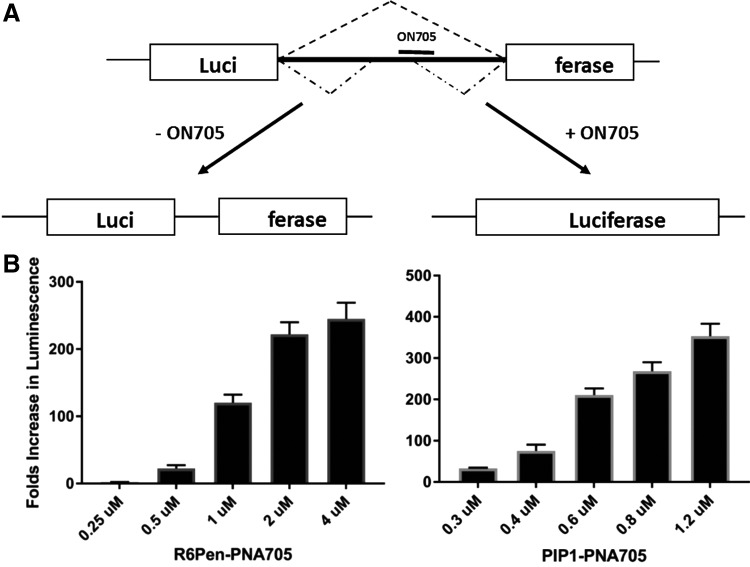
**(A)** Splicing redirection assay by ON 705 to block aberrant splicing in HeLa 705 cells, and **(B)** fold increases in luminescence for R6-Pen-PNA705 and Pip1-PNA705 (note the different scales on the Y axis).

The R_6_-Penetratin peptide was clearly a very useful lead for potential therapeutic development toward steric blocking activity with a PNA cargo. To better assess the potential of this approach for nuclear delivery, in collaboration with Lebleu and colleagues [29], we adopted the HeLa 705 splicing system that was developed by Kole and colleagues based on a mutation discovered in a β-thalassemia patient that resulted in the introduction of a functionally deleterious cryptic splice site [30]. Such activities in the nuclei of cells were particularly suited to fully modified ONs since cleavage, and hence destruction, of the pre-mRNA was not appropriate and instead the ON needed to be bound tightly to block and compete with the action of proteins involved in splicing. The main requirement was the optimal positioning of the ON to hybridize with the pre-mRNA for maximal splicing redirection depending on the particular application. The HeLa 705 system utilizes a steric blocking ON targeted toward a cryptic splice resulting in the removal of the aberrant intron from the luciferase mRNA and subsequent correct protein synthesis that allows for a sensitive readout with a large dynamic range [30] (Fig. 4).

Utilization of a (Lys)_8_-PNA-(Lys) 18-mer targeted to the 705 splice site did not demonstrate splicing correction unless the endosome-disrupting agent chloroquine was added. Subsequent analysis with a N-terminal fluorescently labelled derivative of the (Lys)_8_-PNA-(Lys) suggested that lack of activity was due to ON becoming trapped in endosomal compartments [31]. By contrast, under identical conditions, the use of R_6_-Penetratin (R6-Pen) peptide conjugated to the same 18-mer PNA demonstrated upregulation of luciferase in a luminescence assay of 10^5^-fold higher compared to unconjugated PNA705 [29]. The addition of the endosomal release agent chloroquine to R6Pen-PNA705 increased splicing in the HeLa705 cells two to threefold, suggesting that even with this better CPP most of the R6Pen-PNA705 was still trapped in endosomal compartments. However, the activity level in the absence of chloroquine was higher than that seen for an 8-Arg peptide (R-Ahx-R)_4_-conjugate of PMO and a conjugate of PNA with the same peptide (Arzumanov, Gait, *et al.* unpublished results).

These results led to the use of R_6_-Penetratin as the starting point for design of peptides, which might be suitable as a cell and *in vivo* tissue delivery agent of PNA and later PMO ONs.

## CPP Conjugates of PNA for Exon Skipping in DMD Cells and as *In Vivo* Delivery Agents

In 2007 the Gait laboratory in Cambridge and the Wood laboratory in Oxford began what turned out to be an extremely fruitful collaboration. The HeLa pLuc705 system was used to develop a series of modified R_6_-Penetratin peptides that were more stable to protease digestion in mouse serum, called PNA internalization peptides (Pip) (Fig. 2), which were conjugated to an 18-mer PNA705 model ON. First we showed that introduction of an R-X-R motif (X = aminohexanoic acid, as had been used previously [29]) and shortening of the hydrophobic region resulted in a peptide (Pip1) that conferred threefold higher splicing higher activity compared to R_6_-Penetratin (Fig. 4) and greater than 10-fold higher than both the previously reported peptide [R-Ahx-R]_4_, as well as unconjugated 18-mer PNA705 (data not shown). We showed that Pip1–PNA705 and other Pip conjugates are internalized in HeLa cells by an energy-dependent mechanism and that the predominant pathway of cell uptake of biologically active conjugate is *via* clathrin-dependent endocytosis [32].

**FIG. 5.**
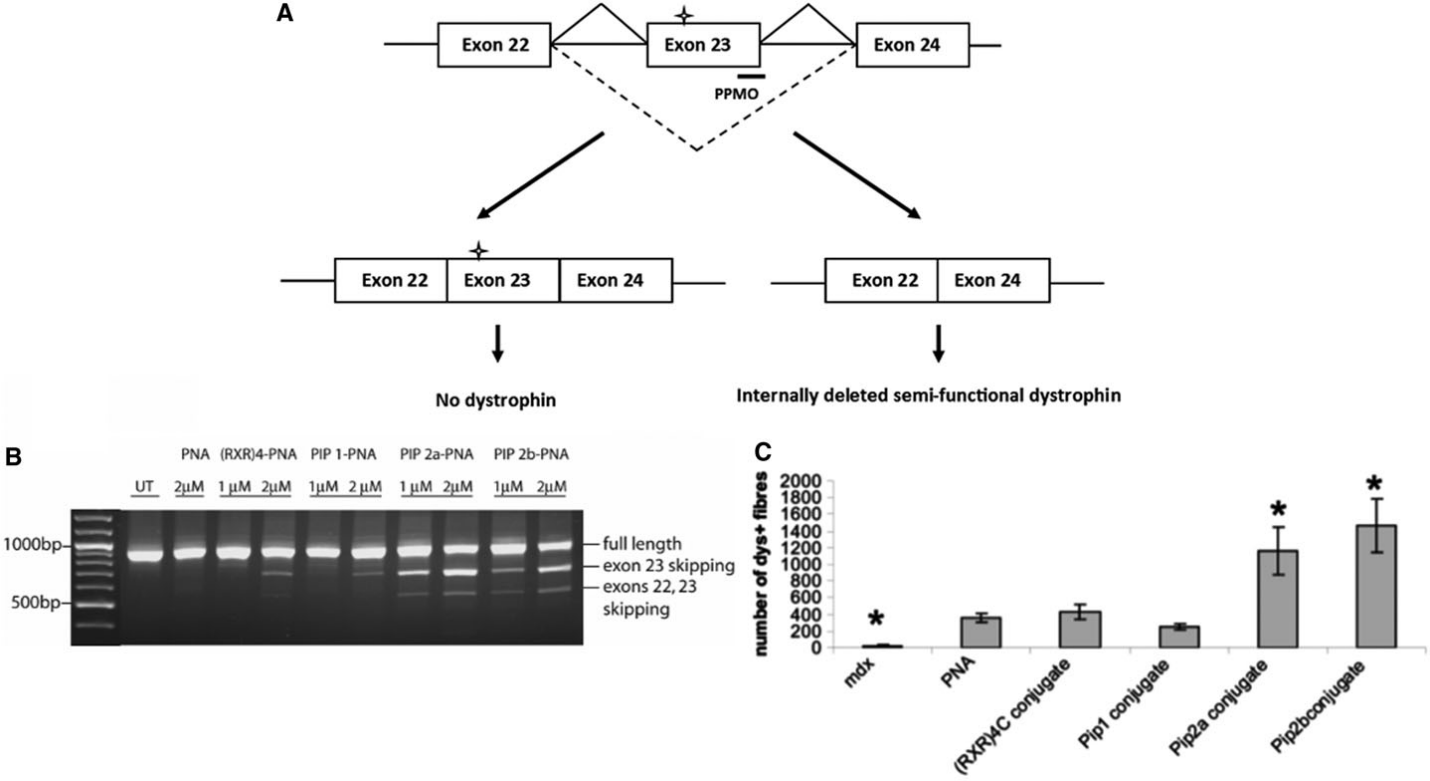
**(A)** The exon skipping assay using PNA ONs in *mdx* mouse cells in culture. The *cross* denotes a premature termination signal, introduced as a result of a point mutation in exon 23 of the dystrophin gene, which gives rise to a truncated nonfunctional protein; **(B, C)** intramuscular delivery in the *mdx* mouse showing **(B)** results as seen by RT-PCR for naked PNA, [R-Ahx-R]_4_-PNA, Pip1-PNA, Pip2a-PNA, and Pip2b-PNA showing the increased exon skipping levels for Pip2a and Pip2b-PNAs, and **(C)** number of dystrophin-positive fibers in immunohistochemistry staining for the same conjugates. *Asterisks* refer to the use of five mice rather than three mice for other experiments. Figures reproduced from reference [24].

Exon skipping has been established for some years as a therapeutic opportunity for the treatment of patients with DMD. Most DMD patients have out-of-frame mutations in the *DMD* gene that results in a truncated and nonfunctional dystrophin protein. In some cases, especially within the repeating units of the spectrin-like rod domain, it is possible to induce in-frame deletions using a synthetic ON targeted to redirect splicing around the genomic deletion, so as to produce a semifunctional protein similar to that produced by less severely affected Becker muscular dystrophy patients [33]. In the well-known *mdx* mouse model of DMD, targeted removal of the in-frame exon 23, which contains a premature termination codon, has become a well-established model to demonstrate the therapeutic potential of splice switching. Serum-stabilized Pip2a or Pip2b (Fig. 2) conjugated to a 20-mer PNA (PNADMD) targeting exon 23 demonstrated enhanced exon-skipping activity in *mdx* myotube cultures in the absence of an added transfection agent at concentrations where the naked PNADMD was inactive [24] **(**Fig. 5A**)**.

In further Pip-PNA synthesis and activity studies, Pip2a and Pip2b conjugates to PNA705 were each joined through a stable thioether linkage, and the peptide part was predominantly stable for 1 h in 20% mouse serum (data not shown). Thus these peptides were considered to be sufficiently stable for use *in vivo*. Therefore *in vivo* experiments were carried out such that injection into the *tibialis anterior* muscles of dystrophic *mdx* mice of Pip2a-PNADMD or Pip2b-PNADMD resulted in greater exon skipping as determined by RT-PCR and approximately threefold higher numbers of dystrophin-positive fibers compared to naked PNADMD or the then standard active control peptide-PNADMD conjugate, (R-Ahx-R)_4_-PNADMD **(**Fig. 5B**)** [24].

## Systemic Delivery of Pip-PMO for Treatment of DMD

Quite surprisingly however, when Peptide-PNA was injected systemically by intravenous tail vein injection into *mdx* mice, the levels of dystrophin production were found to be no higher than naked PNA alone (data not shown). No simple reason could be deduced for this failure, but for further systemic studies we decided instead to switch the ON type to PMO (Fig. 1) that was utilized also by Sarepta.

**FIG. 6.**
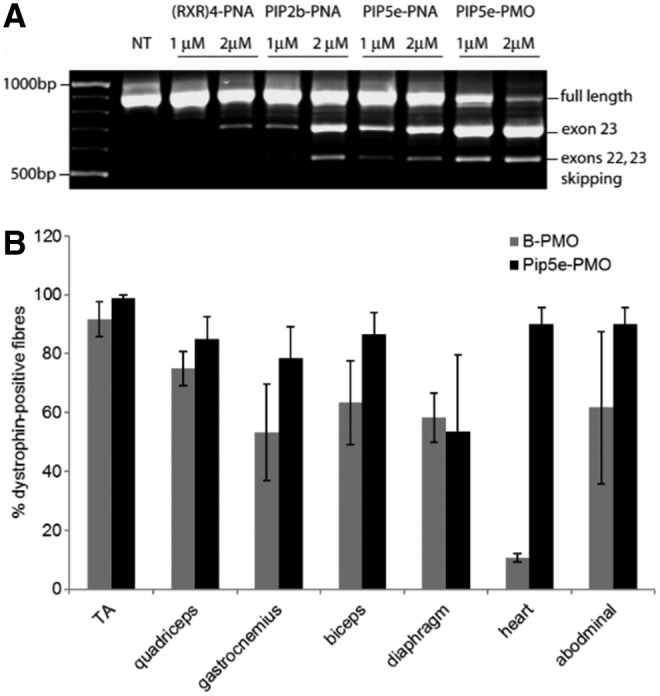
**(A)** Exon skipping in *mdx* cells in culture Pip2b-PNA, Pip5e-PNA, and Pip5e-PMO and **(B)** percentage of dystrophin positive fibers seen for Pip5-PMO conjugates following systemic injections into *mdx* mice, reproduced from reference [34].

Further iterations of Pip design for therapeutic development focused on shortening and simplification of the hydrophobic core region and modification of the cationic arms such that the combination of arginine (R), aminohexanoic acid (Ahx, X), and beta-alanine (B) had a more uniform composition. This Pip5 series of peptides, exemplified by Pip5e (Fig. 2), demonstrated enhanced splice-switching activity *in vitro* compared to previous Pip-PNA iterations (Fig. 6A). A single dose systemic comparator study with the arginine-rich B-PMO (B peptide = RXRRBRRXRRBRXB) demonstrated that Pip5e-PMO had similar efficacy in skeletal muscle, but remarkably enhanced activity in the heart with 25% wild-type dystrophin restoration compared to ∼3% for the B-PMO (Fig. 6B) [34].

**FIG. 7.**
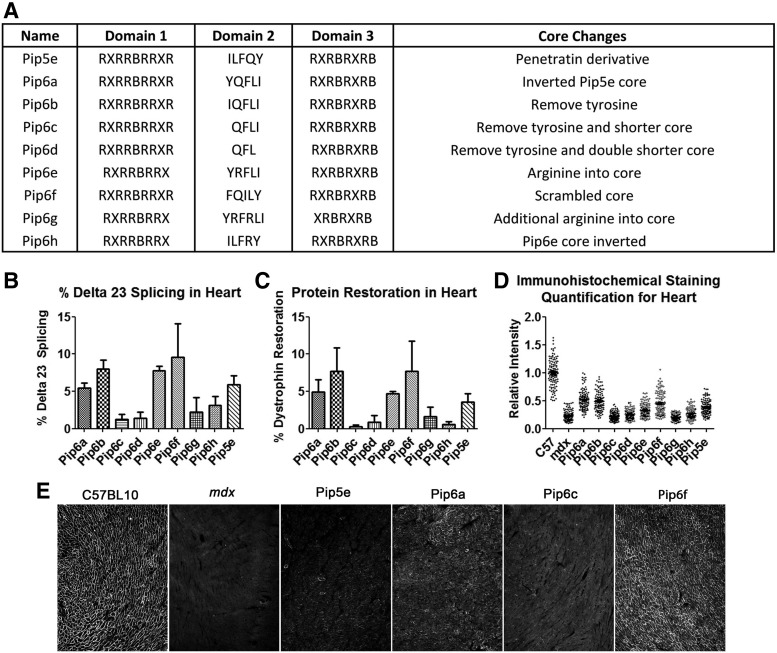
Sequences of Pip6-PMO conjugates, cardiac muscle splicing, and protein restoration following systemic administration of conjugates. A single 12.5 mg/kg systemic injection of peptide-PMO was administered to *mdx* mice, and tissues were harvested 2 weeks later. **(A)** A list of names and sequences, including rationale for synthesis of the peptides used in the study. **(B)** Percentage Δ23 exon skipping determined by q-RT-PCR, **(C)** dystrophin protein restoration determined by western blot, and **(D)** dystrophin immunohistochemistry staining quantified relative to control laminin counter-stain in heart tissue of C57BL10 and *mdx* untreated and *mdx* treated mice. Relative intensity values for each region of interest (120 regions) are plotted. **(E)** Representative dystrophin staining images for C57BL10, *mdx* untreated, and some Pip6 conjugates. Inverted (Pip6a) and scrambled (Pip6f) hydrophobic regions show homogenous dystrophin expression compared to the shortened (Pip6c) peptide. R = arginine; X = aminohexanoic acid; B = beta-alanine. qRT-PCR, quantitative real time-PCR.

This result distinguished the Pip series of peptides from other arginine-rich CPPs and encouraged further series of Pip to be chemically synthesized for use as P-PMO conjugates. First we synthesized derivatives of Pip5e-PMO, consecutively assigned as Pip6-PMOs, and carried out single i.v. dose administration (12.5 mg/kg) into *mdx* mice [35]. These peptide-PMOs comprised alterations to the central 5-amino acid hydrophobic core of the Pip5e peptide **(**Fig. 7A**)** and illustrated that changes to the peptide core sequence, such as inversion (YQFLI) or scrambling (FQILY), resulted in slightly improved dystrophin production, particularly in heart. However, partial deletions within the hydrophobic core (QFLI and QFL) substantially reduced the exon skipping efficiency and dystrophin production (Fig. 7B–D). Our data indicated that the hydrophobic core of the Pip sequences is critical for PMO delivery to the heart and that specific modifications to this region can alter the dystrophin production.

**FIG. 8.**
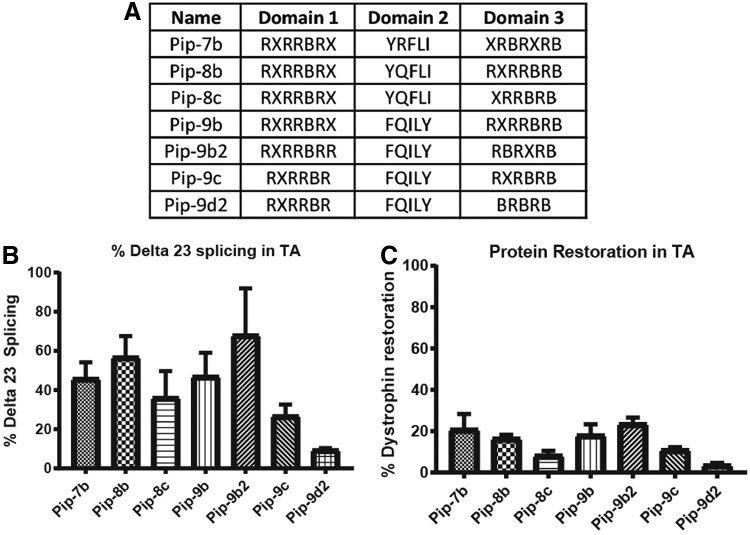
**(A)** Table of exemplar CPP sequences of Pip 7, 8, and 9 series of CPP-PMOs. **(B, C)** Splice-switching activity in *tibialis anterior* of *mdx* mice following a single 12.5 mg/kg intravenous administration as measured by **(B)** quantitative RT-PCR of exon 23 skipping levels and **(C)** dystrophin protein restoration as assessed by western blot. R–arginine; X–aminohexanoic acid; B–beta-alanine.

One of the key challenges for therapeutic success of this class of CPP-PMOs remains the toxicity associated with these peptides. Lethargy in rats following high dose administration of (R-X-R)_4_-XB-PMO has been previously noted [36], and studies in nonhuman primates suggested that the toxicology profile would need to be further improved for clinical development. Our hypothesis was that reduction in the number of arginine residues could confer improved toxicology outcomes, and thus, we sought to reduce the number of Arg residues in the Pip6 series, which contains 10 Arg residues, to between 6 and 9. Thus we developed the Pip 7, 8, and 9 series that were based on the hydrophobic core sequences used in three of the potent Pip6 series, namely Pip6e, Pip6a, and Pip6f, respectively. Carrying out single intravenous dose screening studies in *mdx* mice demonstrated a general pattern of reduced splice-switching activity as Arg residues were removed from the CPP. However the composition of the core region and the precise sequences of the cationic arms still remained the determinant of splicing activity (Fig. 8). Studies are currently underway focusing on the *in vivo* toxicological profile of these compounds and understanding the minimal requirements of CPP composition to maintain robust splice-switching activity while minimizing organ toxicity, especially nephrotoxicity.

## Systemic Delivery of Pip-PMO for Treatment of SMA

Nusinersen (commercialized as spinraza), a wholly 2′-*O*-MOE PS ON (Fig. 1), has been approved for treatment of SMA by direct spinal delivery [6]. SMA is primarily a neurodegenerative disease of the lower motor neurons, and so the spinal cord is the primary tissue for therapeutic disease modification. SMA is caused by the deletion of survival motor neuron 1 gene, *SMN1*. Humans have a second paralog gene of *SMN,* called *SMN2,* which, through a single base change in an exon recognition motif, results in ∼90% of transcripts lacking exon 7 and which results in nonfunctional SMN protein [37]. Thus the number of copies of SMN2 and hence the cumulative levels of the 10% of transcripts that do produce a functional protein is the key determinant of SMA clinical outcome.

**FIG. 9.**
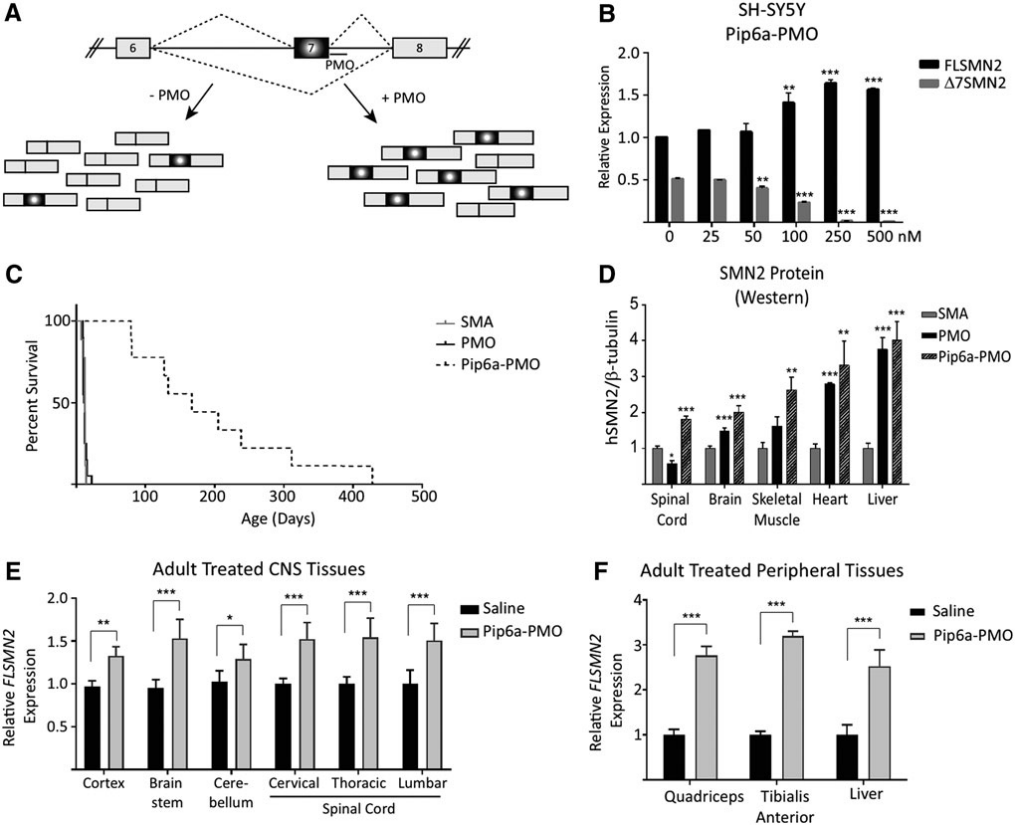
(**A**) Schematic of human *SMN2* alternative splicing of exon 7. The majority of transcripts produced from SMN2 exclude exon 7 (Δ7*SMN2*). Antisense oligonucleotides targeting intron 7 intron splice suppressor element (ISSN1) sterically hinder the binding of *trans*-splice repressor proteins to generate more exon 7-included transcripts (*FLSMN2*). (**B**) Dose-dependent expression of increasing *FLSMN2* and decreasing Δ7*SMN2* transcripts observed in cultured neuroblastoma SH-SY5Y cells following treatment with Pip6a-PMO. Q-PCR expression was normalized to “total *SMN2*” transcripts represented by amplification of exons 2a and 2b. Data are represented as the mean ± SEM. **P ≤ 0.005, ***P ≤ 0.0005, Student's *t*-test relative to untreated expression. (**C**) Direct comparison of systemic PMO versus Pip6a-PMO administration in severe SMA pups. Untreated SMA pups survive a median of 12 days before reaching their humane end point. At 10 μg/g, PMO on its own did not improve survival (median 11.5 days), while 10 μg/g Pip6a-PMO significantly enhanced survival (mean of 196.4 ± 114.9; median 167 days) (*P* ≤ 0.0001 Log-rank Mante-Cox). (**D**) Survival reflective of expression of SMN2 protein in spinal cord, brain, and skeletal muscle 7 days postadministration. Protein analyzed by western blots for human SMN and mouse β-tubulin. Data represented as mean ± SEM. *P ≤ 0.05, **P ≤ 0.01, ***P ≤ 0.001 Student's *t*-test in comparison to untreated SMA mice. **(E, F)** Pip6a-PMO administration into unaffected adult mice harboring the human *SMN2* allele (Smn1^tm1Hung/WT^; SMN2^tg/tg^). Tissues from the (**E**) brain and spinal cord and (**F**) peripheral skeletal muscles and liver were harvested 7 days post 18 mg/kg Pip6a-PMO administration. In all tissues, *FLSMN2* expression was significantly increased over saline treated mice. Data represented by mean ± SEM. *P ≤ 0.005, **P ≤ 0.001, ***P ≤ 0.0001, Student's *t*-test in comparison to saline treated mice [43]. SMA, spinal muscular atrophy.

In SMA, multiple tissue and organs are affected by the low levels of SMN protein (reviewed in [38]). Heterogeneity of phenotype between patients is directly linked to copy numbers of *SMN2* and thereby the levels of SMN protein [39]. Antisense ONs are used to sterically block a splicing repressor motif within intron 7 and thereby increase the exon 7 included transcripts and hence SMN protein expression (Fig. 9A) [40,41]. Unlike the dystrophin gene, a single ON targeted to a single site in the gene for SMN is able to provide treatment for nearly 100% of patients. While direct injection into the central nervous system by lumber puncture is the clinical route of administration for spinraza, seminal work from Adrian Krainer's group showed increased efficacy and survival when the drug was delivered systemically to neonatal SMA mice rather than by direct intracerebroventricular treatment of the central nervous system (CNS) [42]. This suggests that treatment with an effective systemic delivery agent for an ON may have an improved benefit for patients.

Following demonstration that Pip6a-PMO targeted to the repressor sequence at the end of intron 7 increased exon inclusion dose-dependently in cells (Fig. 9B), we therefore started treatment of severe SMA pups and generated biodistribution studies in adult mice using a Pip6A conjugated PMO as a potential efficacious systemic therapeutic [43]. Type I SMA pups normally survive between 7 and 12 days of age. Dosing with Pip6a-PMO at 10 μg/g yielded a median survival of 167 days, a similar survival length to animals given two 80 μg/g doses of nusinersen [42], whereas PMO alone resulted in a median survival of only 12 days (Fig. 9C). SMN protein as judged by western blotting was increased in all tissues assayed 7 days postadministration (Fig. 9D). In unaffected adult mice harboring the human *SMN2* allele, both exon inclusion (Fig. 9E) and SMN protein (Fig. 9F) were increased significantly over saline treated mice when injected with 18 mg/kg Pip6a-PMO and tissues harvested 7 days postadministration.

During observation of the biodistribution of Pip6a-PMO in treated adult mice we were pleased to discover that the Pip6a delivered PMO not only to the skeletal muscle and heart but also to the brain and spinal cord. This was evidenced through upregulation of SMN expression in brain and spinal cord (Fig. 9D), as well as through labelled Pip6a-PMO expression being observed in the brain (data not shown). We have also found that other peptide-PMOs (P-PMOs) are capable of reaching the brain and spinal cord [44]. Now that we know that the Pip series is capable of transporting PMO into the CNS, in mice at least, we have continued with other P-PMOs to further increase this effect while simultaneously work is proceeding to enhance the safety and tolerability to develop clinically relevant peptides (patent application submitted).

## The Future of P-PMO for Treatment of Neuromuscular and Neurodegenerative Diseases

Our extensive studies over several years of some 40 or 50 Pip as covalent PMO conjugates have demonstrated how substantial increases in splice redirecting activity (exon skipping and exon inclusion) are obtained when such Arg-rich peptides are used as cell and *in vivo* delivery agents. Furthermore, by use of the *mdx* mouse model of DMD, we have been able to obtain some insights into how Pip sequence variation affects activities. This has led us into exploration of the use of CPP-PMO conjugates in other neuromuscular diseases through use of alternative mouse models, as well as development of further series of peptides as PMO carriers.

For example, we have recently been studying the effects of Pip6a-PMO in HSA-LR mice, a model of the DM1 disease type in type 1 myotonic dystrophy. In this study we have found that Pip6a-PMO targeting the CUG repeats at the 3′ end of the gene for dystrophin myotonica protein kinase (DMPK), when injected intravenously into HSA-LR mice, corrects splicing defects in the mice, normalizes the global transcriptome at both expression and splicing levels, normalizes the DM1 specific phenotype, namely myotonia, and corrects DM1-specific molecular symptoms in DM1 muscle cells (data not shown). Work is now under way in development of newer CPPs that as conjugates of PMO retain efficacy, but with reduced toxicological measures, which may be suitable drug candidates for treatment of DM1 disease.

In the case of DMD, we have been exploring the use of several new series of Arg-rich CPPs based on the original Pip series. In this study we have not only studied how further sequence modulation affects the exon skipping activity and dystrophin production in the *mdx* mouse model of disease but also measured parameters associated with nephrotoxicity, which is the primary toxicological outcome of this class of compounds. New peptide leads as PMO conjugates are now emerging, and it is hoped that these will lead in due course to a clinical trial for DMD treatment.

A second exciting development is the discovery that Pip6a and another Arg-rich peptide, upon systemic injection, are able to carry a PMO across the blood-brain barrier (BBB) and into brain and spinal cord of mouse models of SMA and lead to both exon inclusion within *SMN2*, as well as phenotypic enhancements [43,44]. This opens up future opportunities with other neuromuscular diseases and many neurodegenerative disorders that could benefit from P-PMO systemic treatment. The promise of therapeutic ON technology envisaged in the 1970s and 1980s is truly coming to fruition, not only in the already well-advanced RNase-H-dependent antisense strategies but also now in the use of steric blocking ONs and their delivery by new classes of Arg-rich CPPs. The power of such platform technologies to enhance systemic delivery of these potent effectors is yet to be realized, but remains an exciting prospect for the antisense therapeutic field and patients alike.

## References

[1] Lundin KE, Gissberg O and Smith CI (2015). Oligonucleotide therapies: the past and the present. Hum Gene Ther 26:475–485 26160334 10.1089/hum.2015.070 PMC4554547

[2] Shen X and Corey DR (2018). Chemistry, mechanism and clinical status of antisense oligonucleotides and duplex RNAs. Nucleic Acids Res 46:1584–1600 29240946 10.1093/nar/gkx1239 PMC5829639

[3] Crooke ST (2017). Molecular mechanisms of antisense oligonucleotides. Nucleic Acid Ther 27:70–77 28080221 10.1089/nat.2016.0656 PMC5372764

[4] Järver P, O'Donovan L and Gait MJ (2014). A chemical view of oligonucleotides for exon skipping and related drug applications. Nucleic Acid Ther 24:37–47 24171481 10.1089/nat.2013.0454 PMC3923385

[5] Aartsma-Rus A, Straub V, Hemmings R, Haas M, Schlosser-Weber G, Stoyanova-Beninska V, Mercuri E, Muntoni F, Sepodes B, Vroom E, Balabanov P (2017). Development of exon skipping therapies for Duchenne Muscular Dystrophy: a critical review and a perspective on the outstanding issues. Nucleic Acid Ther 27:251–259 28796573 10.1089/nat.2017.0682 PMC5649120

[6] Chiriboga CA, Swoboda KJ, Darras BT, Iannaccone ST, Montes J, De Vivo DC, Norris DA, Bennett CF and Bishop KM (2016). Results from a phase 1 study of nusinersen (ISIS-SMNRx) in children with spinal muscular atrophy. Neurology 86:892–897 10.1212/WNL.0000000000002445 PMC4782111 26865511

[7] Monia BP, Lesnik EA, Gonzalez C, Lima WF, McGee D, Guinosso CJ, Kawasaki AM, Cook PD and Freier SM (1993). Evaluation of 2′-modified oligonucleotides containing 2′-deoxy gaps as antisense inhibitors of gene expression. J Biol Chem 268:14514–14522 8390996

[8] Liang XH, Sun H, Shen W and Crooke ST (2015). Identification and characterization of intracellular proteins that bind oligonucleotides with phosphorothioate linkages. Nucleic Acids Res 43:2927–2945 25712094 10.1093/nar/gkv143 PMC4357732

[9] Weidner DA, Valdez BC, Henning D, Greenberg S and Busch H (1995). Phosphorothioate oligonucleotides bind in a non sequence-specific manner to the nucleolar protein C23/nucleolin. FEBS Letts 366:146–150 7789533 10.1016/0014-5793(95)00517-d

[10] Foster DJ, Brown CR, Shaikh S, Trapp C, Schlegel MK, Qian K, Sehgal A, Rajeev KG, Jadhav V, et al. (2018). Advanced siRNA designs further improve in vivo performance of GalNAc-siRNA conjugates. Mol Ther 26:708–717 29456020 10.1016/j.ymthe.2017.12.021 PMC5910670

[11] Graham MJ, Lee RG, Brandt TA, Tai LJ, Fu W, Peralta R, Yu R, Hurh E, Paz E, et al. (2017). Cardiovascular and metabolic effects of ANGPTL3 antisense oligonucleotides. New Engl J Med 377:222–232 28538111 10.1056/NEJMoa1701329

[12] McClorey G and Banerjee S (2018). Cell-penetrating peptides to enhance delivery of oligonucleotide-based therapeutics. Biomedicines 6:51 10.3390/biomedicines6020051 PMC6027240 29734750

[13] Gait MJ and Sheppard RC (1976). A polyamide support for oligonucleotide synthesis. J Am Chem Soc 98:8514–8516

[14] Sproat BS and Gait MJ (1984). Solid-phase synthesis of oligodeoxyribonucleotides by the phosphotriester method. In: Oligonucleotide Synthesis: A Practical Approach. Gait MJ, ed. IRL Press, Oxford, pp 83–114

[15] Beaucage SL and Caruthers MH (1981). Deoxynucleoside phosphoramidites-A new class of key intermediates for deoxypolynucleotide synthesis. Tetrahedron Lett 22:1859–1862

[16] Zamecnik PC and Stephenson ML (1978). Inhibition of Rous sarcoma virus replication and cell transformation by a specific oligodeoxynucleotide. Proc Natl Acad Sci U S A 75:280–284 75545 10.1073/pnas.75.1.280 PMC411230

[17] Eckstein F (2014). Phosphorothioates, essential components of therapeutic oligonucleotides. Nucleic Acid Ther 24:374–387 25353652 10.1089/nat.2014.0506

[18] Baker BF, Lot SS, Condon TP, Cheng-Flournoy S, Lesnik EA, Sasmor HM and Bennett CF (1997). 2′-O-(2-methoxy)ethyl-modified anti-intercellular adhesion molecule 1 (ICAM-1) oligonucleotides selectively increase the ICAM-1 mRNA level and inhibit formation of the ICAM-1 translation initiation complex in human umbilical vein endothelial cells. J Biol Chem 272:11994–12000 9115264 10.1074/jbc.272.18.11994

[19] Dias N, Dheur S, Nielsen PE, Gryaznov S, Van Aerschot A, Herdewijn P, Hélène C and Saison-Behmoaras TE (1999). Antisense PNA tridecamers targeted to the coding region of Ha-ras mRNA arrest polypeptide chain elongation. J Mol Biol 294:403–416 10610767 10.1006/jmbi.1999.3277

[20] Dingwall C, Ernberg I, Gait MJ, Green SM, Heaphy S, Karn J, Lowe AD, Singh M and Skinner MA (1990). HIV-1 tat protein stimulates transcription by binding to a U-rich bulge in the stem of the TAR RNA structure. EMBO J 9:4145–4153 2249668 10.1002/j.1460-2075.1990.tb07637.x PMC552188

[21] Arzumanov A, Walsh AP, Rajwanshi VK, Kumar R, Wengel J and Gait MJ (2001). Inhibition of HIV-1 Tat-dependent trans activation by steric block chimeric 2’-O-methyl/LNA oligoribonucleotides Tat. Biochemistry 40:14645–14654 10.1021/bi011279e 11724578

[22] Ivanova GD, Reigadas S, Ittig D, Arzumanov A, Andreola ML, Leumann C, Toulmé JJ and Gait MJ (2007). Tricyclo-DNA containing oligonucleotides as steric block inhibitors of Human Immunodeficiency Virus Type 1 Tat-dependent trans-activation and HIV-1 infectivity. Oligonucleotides 17:54–65 17461763 10.1089/oli.2006.0046

[23] Langel U (2002). Cell Penetrating Peptides: Processes and Applications. CRC Press, Boca Raton

[24] Ivanova GD, Arzumanov A, Abes R, Yin H, Wood MJ, Lebleu B and Gait MJ (2008). Improved cell-penetrating peptide-PNA conjugates for splicing redirection in HeLa cells and exon skipping in *mdx* mouse muscle. Nucleic Acids Res 36:6418–6428 18842625 10.1093/nar/gkn671 PMC2582604

[25] Gait MJ (2003). Peptide-mediated cellular delivery of antisense oligonucleotides and their analogues. Cell Mol Life Sci 60:844–853 12827274 10.1007/s00018-003-3044-5 PMC11138796

[26] Richard JP, Melikov K, Vivès E, Ramos C, Verbeure B, Gait MJ, Chernomordik LV and Lebleu B (2003). Cell-penetrating peptides. A re-evaluation of the mechanism of cellular uptake. J Biol Chem 278:585–590 12411431 10.1074/jbc.M209548200

[27] Turner JJ, Arzumanov AA and Gait MJ (2005). Synthesis, cellular uptake and HIV-1 Tat-dependent trans-activation inhibition activity of oligonucleotide analogues disulphide-conjugated to cell-penetrating peptides. Nucleic Acids Res 33:27–42 15640444 10.1093/nar/gki142 PMC546131

[28] Turner JJ, Ivanova GD, Verbeure B, Williams D, Arzumanov AA, Abes S, Lebleu B and Gait MJ (2005). Cell-penetrating peptide conjugates of peptide nucleic acids (PNA) as inhibitors of HIV-1 Tat-dependent trans-activation in cells. Nucleic Acids Res 33:6837–6849 16321967 10.1093/nar/gki991 PMC1301599

[29] Abes S, Moulton HM, Clair P, Prevot P, Youngblood DS, Wu RP, Iversen PL and Lebleu B (2006). Vectorization of morpholino oligomers by the (R-Ahx-R)_4_ peptide allows efficient splicing correction in the absence of endosomolytic agents. J Control Release 116:304–313 17097177 10.1016/j.jconrel.2006.09.011

[30] Kang SH, Cho MJ and Kole R (1998). Up-regulation of luciferase gene expression with antisense oligonucleotides: implications and applications in functional assay development. Biochemistry 37:6235–6239 9572837 10.1021/bi980300h

[31] Abes S, Williams D, Prevot P, Thierry AR, Gait MJ and Lebleu B (2006). Endosome trapping limits the efficiency of splicing correction by PNA-oligolysine conjugates. J Control Release 110:595–604 16377019 10.1016/j.jconrel.2005.10.026

[32] Ivanova GD, Arzumanov AA, Turner JJ, Fabani MM, Abes R, Lebleu B and Gait MJ (2008). RNA targeting in cells by peptide conjugates of peptide nucleic acids (PNA). Collection Symp Ser 10:103–111

[33] Aartsma-Rus A (2012). Overview on DMD exon skipping. In: Exon Skipping. Methods and Protocols. Aartsma-Rus A, ed. Humana Press, New York, pp. 97–116 10.1007/978-1-61779-767-5_7 22454057

[34] Yin H, Saleh AF, Betts C, Camelliti P, Seow Y, Ashraf S, Arzumanov A, Hammond S, Merritt T, Gait MJ, and Wood MJ (2011). Pip5 transduction peptides direct high efficiency oligonucleotide-mediated dystrophin exon skipping in heart and phenotypic correction in mdx mice. Mol Ther 19:1295–1303 10.1038/mt.2011.79 PMC3128823 21505427

[35] Betts C, Saleh AF, Arzumanov AA, Hammond SM, Godfrey C, Coursindel T, Gait MJ and Wood MJ (2012). A new generation of peptide-oligonucleotide conjugates with improved cardiac exon skipping activity for Duchenne muscular dystrophy treatment. Mol Ther Nucleic Acids 1:e38 23344180 10.1038/mtna.2012.30 PMC3438601

[36] Amantana A, Moulton HM, Cate ML, Reddy MT, Whitehead T, Hassinger JN, Youngblood DS and Iversen PL (2007). Pharmacokinetics, biodistribution, stability and toxicity of a cell-penetrating peptide-morpholino oligomer conjugate. Bioconjug Chem 18:1325–1331 17583927 10.1021/bc070060v

[37] Lorson CL, Hahnen E, Androphy EJ and Wirth B (1999). A single nucleotide in the SMN gene regulates splicing and is responsible for spinal muscular atrophy. Proc Natl Acad Sci U S A 96:6307–6311 10339583 10.1073/pnas.96.11.6307 PMC26877

[38] Simone C, Ramirez A, Bucchia M, Rinchetti P, Rideout H, Papadimitriou D, Re DB and Corti S (2016). Is spinal muscular atrophy a disease of the motor neurons only: pathogenesis and therapeutic implications? Cell Mol Life Sci 73:1003–1020 26681261 10.1007/s00018-015-2106-9 PMC4756905

[39] Wirth B (2000). An update of the mutation spectrum of the survival motor neuron gene (SMN1) in autosomal recessive spinal muscular atrophy (SMA). Hum Mutat 15:228–237 10679938 10.1002/(SICI)1098-1004(200003)15:3<228::AID-HUMU3>3.0.CO;2-9

[40] Hua Y, Vickers TA, Baker BF, Bennett CF and Krainer AR (2007). Enhancement of SMN2 exon 7 inclusion by antisense oligonucleotides targeting the exon. PLoS Biol 5:e73 17355180 10.1371/journal.pbio.0050073 PMC1820610

[41] Singh NK, Singh NN, Androphy EJ and Singh RN (2006). Splicing of a critical exon of human Survival Motor Neuron is regulated by a unique silencer element located in the last intron. Mol Cell Biol 26:1333–1346 16449646 10.1128/MCB.26.4.1333-1346.2006 PMC1367187

[42] Hua Y, Sahashi K, Rigo F, Hung G, Horev G, Bennett CF and Krainer AR (2011). Peripheral SMN restoration is essential for long-term rescue of a severe spinal muscular atrophy mouse model. Nature 478:123–126 21979052 10.1038/nature10485 PMC3191865

[43] Hammond SM, Hazell G, Shabanpoor F, Saleh AF, Bowerman M, Sleigh JN, Meijboom KE, Zhou H, Muntoni F, et al. (2016). Systemic peptide-mediated oligonucleotide therapy improves long-term survival in spinal muscular atrophy. Proc Natl Acad Sci U S A 113:10962–10967 27621445 10.1073/pnas.1605731113 PMC5047168

[44] Shabanpoor F, Hammond SM, Abendroth F, Hazell G, Wood MJA and Gait MJ (2017). Identification of a peptide for systemic brain delivery of a morpholino oligonucleotide in mouse models of Spinal Muscular Atrophy. Nucl Acid Ther 27:130–143 10.1089/nat.2016.0652 PMC5467147 28118087

